# Single-versus multiple-inhaler triple therapy in patients with COPD in Spain: a retrospective cohort study comparing adherence, persistence, risk of exacerbations and economic outcomes

**DOI:** 10.3389/fphar.2025.1642470

**Published:** 2025-09-19

**Authors:** M. Asunción González-González, M. Aránzazu Pedrosa-Naudín, Diego Fernández-Lázaro, Isabel Díaz Planelles, F. Javier Álvarez, Eduardo Gutiérrez-Abejón

**Affiliations:** ^1^ Pharmacy Directorate, Castilla y León Health Council, Valladolid, Spain; ^2^ Area of Histology and Neurobiology Research Group, Faculty of Medicine, University of Valladolid, Valladolid, Spain; ^3^ Facultad de Empresa y Comunicación, Universidad Internacional de la Rioja (UNIR), Logroño, Spain; ^4^ Laboratory of Pharmacoepidemiological Research in Primary Care, Health Research Institute of Valladolid (IBioVALL), Valladolid, Spain; ^5^ Area of Pharmacology, Department of Cell Biology, Genetics, Histology and Pharmacology, Faculty of Medicine, University of Valladolid, Valladolid, Spain; ^6^ CEIm of the Valladolid Health Areas, Valladolid, Spain; ^7^ Atención Primaria, Área de Salud de Valladolid Este, Valladolid, Spain

**Keywords:** chronic obstructive pulmonary disease, multiple-inhaler triple therapy, single-inhaler triple therapy, adherence, persistence, exacerbations

## Abstract

This retrospective study aimed to compare the clinical and economic outcomes of single-inhaler triple therapy (SITT) versus multiple-inhaler triple therapy (MITT) in a large cohort of COPD patients. Metrics on adherence, prevalence, and incidence of exacerbations in COPD patients treated with SITT or MITT were analyzed using pharmacy claims data integrated with the Spanish public health database. At the 12-month follow-up, patients in the SITT cohort were significantly more adherent (75.22% vs 70.1%; OR = 1.33), more persistent (64.32% vs 52.4%; HR = 1.56) and had a lower incidence of moderate exacerbations (53.53% vs 64.07%; OR = 0.65) than patients in the MITT cohort. The main predictors associated with lack of persistence were being a naïve patient (HR = 0.55) and moderate exacerbations (HR = 0.85). Furthermore, medication costs were lower for SITT (EUR 909.31 vs EUR 1025.31), demonstrating its cost-effectiveness. Our results suggest that SITT not only may improve adherence and persistence but also contributes to a relevant reduction in the risk of moderate exacerbations. Additionally, SITT offers a more cost-effective alternative for patients with moderate to severe COPD with documented exacerbations, making it a valuable strategy in real-world clinical practice.

## 1 Introduction

Chronic obstructive pulmonary disease (COPD) is a progressive inflammatory disease characterized by severe respiratory symptoms that can cause irreversible airflow limitation (Torpy et al., 2012). COPD has a global prevalence of 10.3% (Adeloye et al., 2022) and is a leading cause of morbidity and mortality, particularly in patients with exacerbations (Global Initiative for Chronic Obstructive and Lung Disease, 2024). In fact, COPD is expected to become the fourth leading cause of death worldwide by 2030 (Mathers and Loncar, 2006).

Treatment for COPD includes inhaled medications such as long-acting β2-agonists (LABA), long-acting muscarinic antagonists (LAMA), and inhaled corticosteroids (ICS). Medications are selected based on symptoms and risk of exacerbations. For patients with moderate to severe disease who continue to experience exacerbations or poor symptom control on dual therapy, triple therapy (LABA/LAMA/ICS) is recommended (Global Initiative for Chronic Obstructive and Lung Disease, 2024). For these patients, triple therapy has been shown to provide clinical benefits over dual therapy, including improved lung function, lower hospitalization rates (Frith et al., 2015), and reduced mortality (Lipson et al., 2020; Rabe et al., 2020).

In patients with COPD, medication adherence is lower than in other chronic diseases, such as diabetes, depression, or hypertension (Rolnick et al., 2013). According to the literature, the rate of non-adherence to inhaled medications ranges from 22% to 93% (Bhattarai et al., 2020). Adherence to treatment is negatively impacted by the use of different inhalation devices, errors in inhalation technique, and complex dosing regimens (Honkoop et al., 2022). In this sense, non-adherence to inhaled medications for COPD has been associated with a worsening of symptoms, an increased risk of exacerbations, a reduced quality of life, increased hospitalization rates, and higher mortality (Świątoniowska et al., 2020). Additionally, non-adherence to inhaled therapy in patients with COPD is associated with poorer economic outcomes (Cushen et al., 2018).

Triple therapy can be administered as single-inhaler triple therapy (SITT) or multiple-inhaler triple therapy (MITT). In this sense, SITT offers a simplified inhalation regimen (Meynell and Capstick, 2018), improving adherence (Mannino et al., 2022; Lin et al., 2023; Bogart et al., 2024) and persistence (Alcázar-Navarrete et al., 2022; Mannino et al., 2022; Deslee et al., 2023; Lin et al., 2023) while also reducing exacerbations and healthcare resource utilization (Alcázar-Navarrete et al., 2022; Bogart et al., 2024).

This study compared the use of SITT versus MITT in a large cohort of COPD patients. It evaluated treatment adherence and persistence, exacerbation prevention, and analyzed cost-effectiveness, providing new insights into the potential advantages of SITT in a real-world setting.

## 2 Materials and methods

### 2.1 Study design and data source

This observational and retrospective cohort study was conducted in Castilla y León, Spain, with a population of 2,327,420 inhabitants (Spanish National Statistics Institute INE, 2024). The study was designed according to the Strengthening the Reporting of Observational Studies in Epidemiology (STROBE) (Von Elm et al., 2008) and the Reporting of Studies Conducted using Observational Routinely collected health Data for Pharmacoepidemiology (RECORD-PE) (Langan et al., 2018) recommendations.

Claims data were obtained from the Pharmaceutical Information System of Castilla y León (CONCYLIA) (Castile and Leon Health Council, 2024). This data source contains primary care prescribing and dispensing data for all patients covered by the Spanish National Health Service, approximately 97% of the population.

Patient data in CONCYLIA are anonymized, so informed consent was not required. This study was approved by the Ethics Committee of the Valladolid Health Areas on October 9, 2024 (reference number PI-24-561-APE).

### 2.2 Study population

Patients with a diagnosis of COPD according to the International Classification of Diseases-10 (ICD-10) (World Health Organization, 2016), ≥40 years of age, and with at least two prescription refills of SITT or MITT between January 1, 2021, and December 31, 2023, were selected. The index date was defined as the date of SITT or MITT initiation. For MITT, the date considered was the first day with an overlapping supply of all MITT components. Supplementary Table S1 shows the SITTs and MITTs available in Spain.

Patients with less than 12 months of follow-up, inconsistent medication records (date of dispensing not available), or who died during the study period were excluded.

Naive patients were defined as those who had not received inhaled medication (ATC subgroups R03A and R03B) 12 months before study entry.

### 2.3 Study variables and definitions

Sociodemographic, clinical and economic data were obtained from CONCYLIA. Sociodemographic data included sex, age, institutionalization, healthcare area and socioeconomic level. Clinical data included type of inhaled medication (dose and dosing regimen), concomitant medication (polypharmacy), multiple prescribers and multiple pharmacies (≥3/year), comorbidities, adherence, persistence at 3, 6 and 12 months, mean persistence (in days), persistence rate and moderate exacerbations (frequency and rate). Economic data included medication costs per patient/year and incremental cost-effectiveness ratio (ICER).

A moderate exacerbation is characterized by administering oral corticosteroids and respiratory antibiotics (ATC subgroups J01AA and J01CA) for COPD diagnosis. A recurrence of the same exacerbation was considered if the interval between exacerbations was less than 4 weeks. Severe exacerbations could not be collected as CONCYLIA does not include data on hospital admissions.

Adherence was measured using the Medication Possession Rate (MPR), calculated as the number of days’ supply during a specified follow-up period (365 days) divided by the number of days from the first dispensing to the end of the follow-up period (Andrade et al., 2006). Adherence was classified according to MPR as follows: none (<20), poor (20-49), moderate (50-79) and adherent (≥80) (Krivoy et al., 2016; Pedrosa-Naudín et al., 2022). The percentage of adherent patients was calculated for each type of therapy.

Persistence was defined as the period between the index date and treatment discontinuation. A gap of >60 days between prescription refills for any component of MITT or SITT was considered a discontinuation. The percentage of persistent patients at 3, 6 and 12 months was calculated. The persistence rate for each patient was calculated as the persistent days divided by the follow-up period (365 days). Drug switching (different ATC codes) within the same type of triple therapy (any component of MITT or SITT) was considered as discontinuation. A dose escalation was considered a continuation of treatment. Sensitivity analyses were performed by modifying the allowed gap between prescription refills from 60 to 90 days.

Medication costs were calculated using the prices listed in the official medicines formulary of the Spanish National Health System (Spanish Ministry of Health, 2025). The mean cost per patient/year was calculated for MITT and SITT. The persistence rate was used to measure the effectiveness of ICER calculation (Romagnoli et al., 2020). All costs were reported in euros using 2023 values. The exchange rates of EUR1 = US$1.105 and EUR1 = £0.8683 were based on the European Central Bank rate on December 29, 2023.

### 2.4 Statistical analysis

Results are presented as means with their standard deviations (SD) or as percentages with their 95% confidence intervals (95% CI), as appropriate. The Kolmogorov-Smirnov test determined the sample’s normality. Student’s t-test for continuous variables and chi-squared test for categorical variables were used to assess differences between groups.

Binary logistic regression was used to analyze the factors influencing adherence. Treatment persistence and duration were analyzed using Kaplan-Meier survival analysis and the log-rank test to compare both groups. The absence of an event (treatment discontinuation) resulted in data censoring. Cox proportional hazards regression was used to assess persistence at 12 months. Univariate and multivariate analyses were performed. All study variables were included in the univariate analysis. Variables with p ≤ 0.05 in the univariate analysis were excluded from the multivariate analysis.

SPSS version 24.0 (SPSS Inc, Chicago, IL) was used for statistical analysis. Statistical significance was determined at p ≤ 0.05.

## 3 Results

**FIGURE 1:**
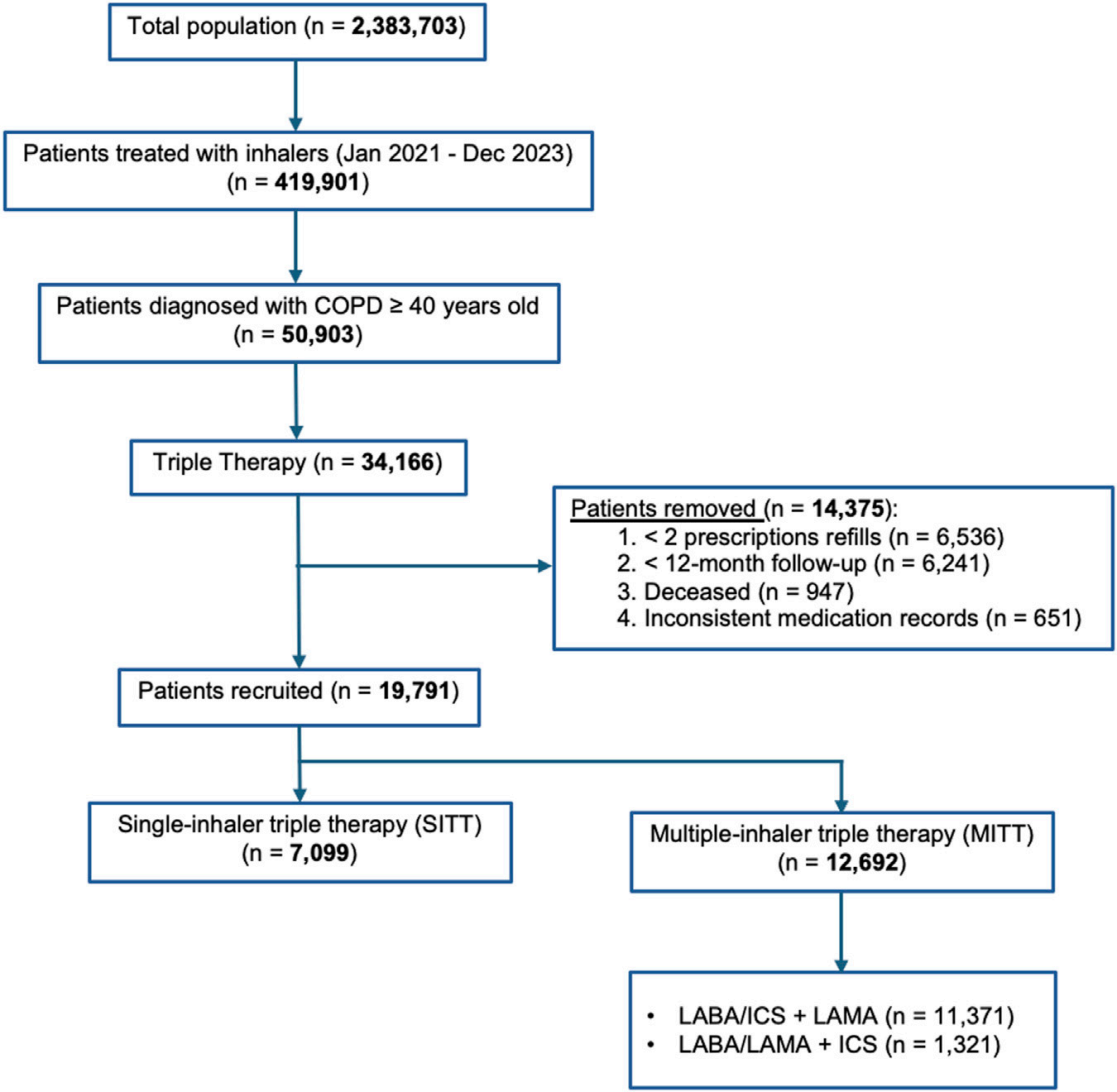
Flowchart of the study population (MITT, multiple-inhaler triple therapy; SITT, single-inhaler triple therapy; LABA, long-acting β2-agonists; LAMA, long-acting muscarinic antagonists; ICS, inhaled corticosteroids).

The study population included 7,099 patients in the SITT cohort and 12692 in the MITT cohort. In the MITT cohort, 90% of patients received a LABA/ICS plus LAMA combination (Figure 1). The mean age was 74.71 ± 11.98 years; 63% were male, and 9.5% were naïve patients (Supplementary Table S2).

The incidence of moderate exacerbations was lower in the SITT cohort (53.53%) than in the MITT cohort (64.07%). A decreased risk was also observed in the SITT group (OR = 0.65; p = 0.001) (Table 1). Patients in the SITT cohort required less dose escalation than those in the MITT cohort (6.55% vs 11.84%; p = 0.001) (Supplementary Table S2).

**TABLE 1:** Adherence, persistence and moderate exacerbations during 12-month follow-up.

		Total	MITT	SITT	p
N		19791	12692	7099	
Adherence % (95% IC)					
MPR (mean ± SD)		84.74 ± 16.79	84.15 ± 16.55	85.80 ± 17.17	0.001
Adherence prevalence		71.94 (71.31-72.56)	70.1 (69.3-70.9)	75.22 (74.22-76.23)	0.001
Adherence level					
	None (<20)	0.52 (0.42-0.62)	0.39 (0.28-0.5)	0.73 (0.53-0.93)	0.001
	Poor (20-49)	4.82 (4.52-5.11)	4.88 (4.5-5.25)	4.7 (4.21-5.2)	
	Moderate (50-79)	22.73 (22.15-23.32)	24.63 (23.88-25.38)	19.34 (18.42-20.26)	
OR for adherence^a^ (95% CI)				1.33 (1.24-1.42)	0.001
Persistence % (95% CI)					
	3 months	82.39 (81.86-82.92)	79.96 (79.26-80.65)	86.73 (85.94-87.52)	0.001
	6 months	68.31 (67.67-68.96)	64.44 (63.61-65.27)	75.24 (74.23-76.24)	0.001
	12 months	56.68 (55.99-57.37)	52.4 (51.53-53.27)	64.32 (63.2-65.43)	0.001
Treatment persistence (days) (mean ± SD)		261.85 ± 129.61	249.99 ± 132.91	283.05 ± 120.63	0.001
HR for persistence^b^ (95% CI)				1.56 (1.49-1.64)	0.001
Moderate exacerbations % (95% IC)					
Patients with moderate exacecerbations		60.29 (59.61-60.97)	64.07 (63.24-64.91)	53.53 (52.37-54.69)	0.001
	1 exacerbations	45.5 (44.81-46.19)	48.43 (47.56-49.3)	40.27 (39.13-41.41)	0.001
	≥ 2 exacerbations	14.79 (14.3-15.28)	15.64 (15.01-16.27)	13.26 (12.47-14.05)	
No. Exacerbations (mean ± SD)		0.82 ± 0.81	0.85 ± 0.83	0.78 ± 0.7	0.001
OR for moderate exacerbations^a^ (95% CI)				0.65 (0.61-0.69)	0.001

^a^ Reference group for logistic regression model: SITT (multivariate analysis).

^b^ Reference group for Cox proportional hazards model: SITT (multivariate analysis).

MITT, multiple-inhaler triple therapy; SITT, single-inhaler triple therapy; CI, confidence interval; SD, standard deviation; OR, odds ratio; HR, hazard ratio.

The proportion of adherent patients was higher in the SITT cohort than in the MITT cohort (75.22% vs 70.1%; p = 0.001). Patients in the SITT cohort were more likely to be adherent than those in the MITT cohort (OR = 1.33; p = 0.001) (Table 1).

**FIGURE 2:**
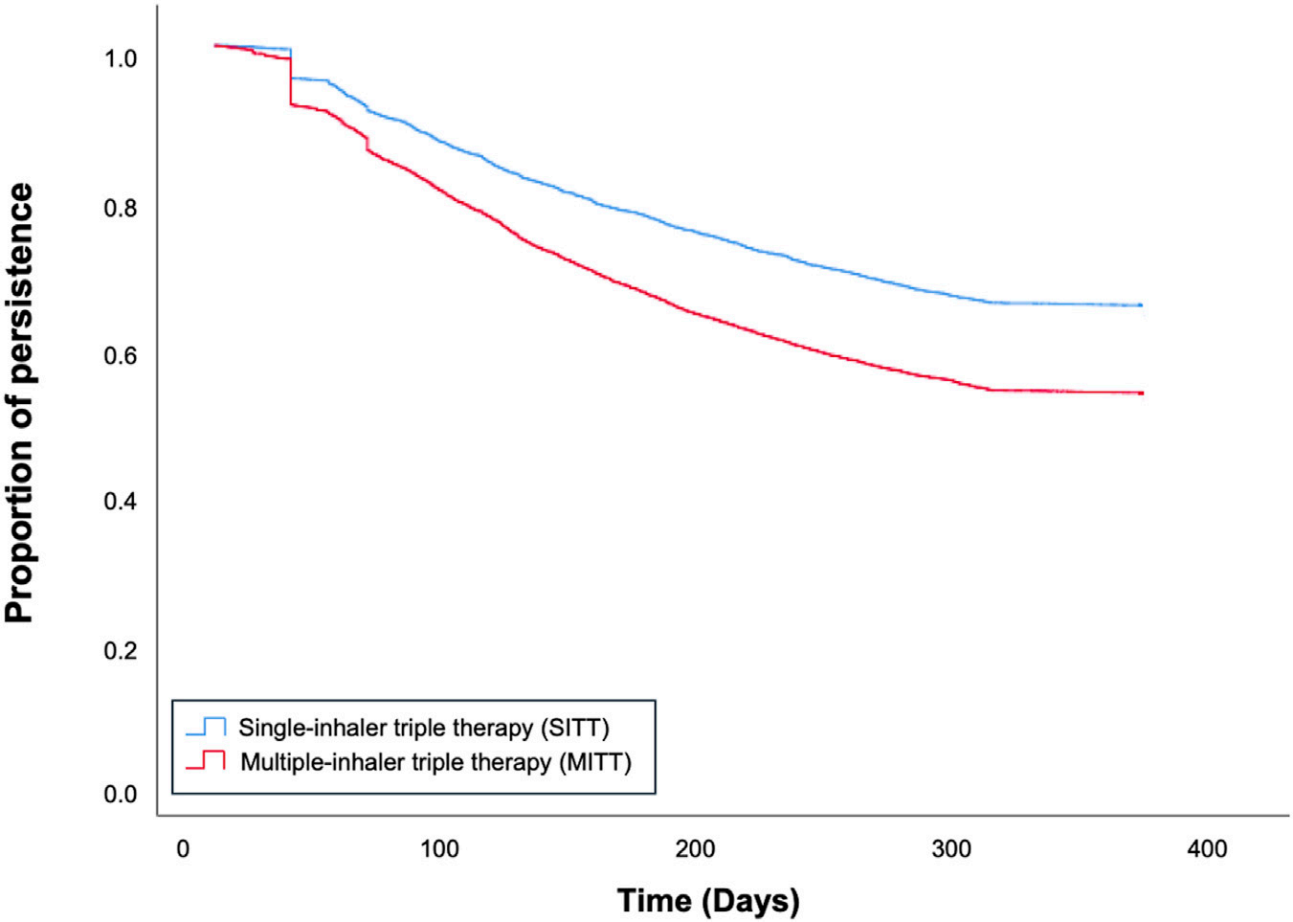
Kaplan-Meier curve for treatment persistence in patients with COPD on single-inhaler triple therapy (SITT) and multiple-inhaler triple therapy (MITT) in Spain.

Patients in the SITT group were more persistent than those in the MITT group at 3 (86.73% vs 79.96%; p = 0.001), 6 (75.24% vs 64.44%; p = 0.001), and 12 months (64.32% vs 52.4%: p = 0.001). Furthermore, mean treatment persistence was higher in the SITT cohort than in the MITT cohort (283.05 ± 120.63 vs 249.99 ± 132.91; p = 0.001) (Table 1; Figure 2).

At the 12-month follow-up, persistence was favored in the SITT cohort (HR = 1.56; p = 0.001) (Table 1). Modifying the gap between prescription refills from 60 to 90 days had comparable results (Supplementary Table S3).

Being a naïve patient was associated with a lack of persistence (HR = 0.55; p = 0.001). Only 46.63% and 30.65% of naïve patients in the SITT and MITT cohorts were persistent, with mean treatment persistence decreasing to 237.12 ± 131.77 and 188.74 ± 132.45 days, respectively. Moderate exacerbations were strongly associated with a lack of persistence (HR = 0.85; p = 0.001). Other associated factors were female sex, institutionalization, multiple prescribers, polypharmacy, and diagnoses of asthma and depression (Table 2; Supplementary Figure S1).

**TABLE 2:** Predictors of persistence in patients with COPD on triple inhaled therapy in Spain.

Variables	Univariate analysis		Multivariate analysis	
	HR (95% CI)	p	HR (95% CI)	p
Type of Inhalation Therapy (SITT)	1.48 (1.41-1.55)	0.001	1.56 (1.49-1.64)	0.001
Sociodemographic characteristics				
*Sex (female)*	0.75 (0.72-0.78)	0.001	0.85 (0.81-0.89)	0.001
*Age*	0.99 (0.99-0.99)	0.001	1,01 (0.97-1.05)	0.087
*Instituzionalized (Yes)*	0.73 (0.67-0.79)	0.001	0.72 (0.66-0.79)	0.001
*Healthcare area (Urban)*	0.97 (0.93-1.01)	0.145		
*Socioeconomical level*				
*< 18000 €/year*	1 (reference)			
*18000-100000€/year*	1.1 (0.76-1.58)	0.61		
*≥ 100000 €/year*	1.17 (0.81-1.68)	0.41		
Clinical characteristics				
*Naive patient (Yes)*	0.57 (0.54-0.61)	0.001	0.55 (0.52-0.59)	0.001
*Multiple prescribers (Yes)*	0.8 (0.76-0.84)	0.001	0.83 (0.79-0.87)	0.001
*Multiple pharmacies (Yes)*	0.97 (0.93-1.01)	0.157		
*Polypharmacy (Yes)*	0.72 (0.65-0.81)	0.001	0.86 (0.76-0.97)	0.015
*Dose escalation (Yes)*	0.83 (0.78-0.89)	0.001	0.95 (0.89-1.02)	0.151
*Moderate exacerbations (Yes)*	0.77 (0.73-0.8)	0.001	0.85 (0.81-0.89)	0.001
Comorbidities				
*Hypertension (Yes)*	1.06 (1.01-1.1)	0.015	1 (0.95-1.05)	0.99
*Dyslipidemia (Yes)*	1.03 (0.99-1.08)	0.151		
*Anxiety (Yes)*	0.87 (0.84-0.91)	0.001	0.98 (0.94-1.03)	0.474
*Asthma (Yes)*	0.79 (0.76-0.83)	0.001	0.92 (0.87-0.96)	0.001
*Depression (Yes)*	0.82 (0.78-0.86)	0.001	0.91 (0.87-0.96)	0.001
*Diabetes Mellitus (Yes)*	1.03 (0.98-1.08)	0.316		
*Ischaemic heart disease (Yes)*	1.01 (0.96-1.07)	0.705		
*Heart failure (Yes)*	0.98 (0.93-1.04)	0.497		
*Psychotic illness (Yes)*	0.87 (0.81-0.93)	0.001	0.99 (0.92-1.07)	0.798
*Dementia (Yes)*	0.99 (0.83-1.19)	0.926		

SITT, single-inhaler triple therapy; CI, confidence interval; HR, hazard ratio.

Persistence rates were 0.78 ± 0.33 for the SITT cohort and 0.68 ± 0.36 for the MITT cohort. The mean costs of SITT and MITT were EUR 909.31 ± 13.86 and EUR 1,025.31 ± 96.05 per patient per year, respectively. The ICER of SITT versus MITT ranged from EUR −1,160 to EUR −1,450 per persistence rate, depending on the gap between prescription refills (60 or 90 days).

## 4 Discussion

Our findings show that patients in the SITT cohort were more adherent than those in the MITT cohort (75% vs 70%). Furthermore, patients in the SITT group showed greater persistence, with a rate more than 10% higher after 12 months of treatment. The main predisposing factors for lack of persistence were identified as being a naive patient, having multiple prescribers, being female, and experiencing moderate exacerbations. Additionally, moderate exacerbations occurred 10% more frequently among patients in the MITT cohort.

Our patients’ baseline characteristics are like those in other European (Alcázar-Navarrete et al., 2022; Deslee et al., 2023), American (Mannino et al., 2022; Bogart et al., 2024), and Chinese (Lin et al., 2023) studies. The presence of almost 10% of naïve patients is relevant and similar to another European study (Deslee et al., 2023). However, the Global Initiative for Chronic Obstructive Lung Disease (GOLD) report (Global Initiative for Chronic Obstructive and Lung Disease, 2024) recommends, albeit with reservations, triple therapy for naïve patients with a blood eosinophil count ≥300 cells/μl. As in other observational studies (Alcázar-Navarrete et al., 2022; Mannino et al., 2022; Deslee et al., 2023), patients with a concomitant diagnosis of asthma were not excluded, in contrast to other more restrictive studies (Lin et al., 2023).

Previous studies show higher adherence in the SITT group than in the MITT group (Mannino et al., 2022; Lin et al., 2023; Bogart et al., 2024), except for a French study (Deslee et al., 2023), although its results are not significant. For the SITT cohort, results ranged from 26% (Bogart et al., 2024) to 76.8% (Mannino et al., 2022) of adherent patients, and for the MITT cohort, from 13.1% (Bogart et al., 2024) to 65.5% (Lin et al., 2023). Our results are like the maximum adherence values, although the comparison should be made cautiously because the other studies used the proportion of days covered (PDC) (Mannino et al., 2022; Lin et al., 2023; Bogart et al., 2024) as a measure of adherence instead of MPR. As in other studies (Alcázar-Navarrete et al., 2022; Mannino et al., 2022; Deslee et al., 2023; Lin et al., 2023; Bogart et al., 2024), higher persistence was observed in the SITT cohort than in the MITT cohort. Our findings were like those of other studies that defined discontinuation with a gap between prescription refills of 60 days (Deslee et al., 2023; Lin et al., 2023; Bogart et al., 2024) and higher than those that used a gap of 30 days (Mannino et al., 2022).

The incidence and risk of moderate exacerbations were lower in the SITT cohort than in the MITT cohort, consistent with other published results (Alcázar-Navarrete et al., 2022; Lin et al., 2023). In addition, our results showed a direct relationship between moderate exacerbations and lack of persistence. On the other hand, this result may have been influenced by the higher proportion of patients with concomitant asthma in the MITT group than in the SITT group.

Additionally, the MITT cohort required almost twice dose escalation as the SITT cohort. A greater need for dose escalation, lower persistence, and a higher incidence of moderate exacerbations may suggest that the MITT cohort has lower treatment efficacy than the SITT cohort.

Being a naïve patient was associated with a 45% decrease in persistence, reducing the mean treatment persistence by 50 days in both cohorts. Other predictors of decreased persistence were a diagnosis of asthma (8%) and depression (9%). Another study looking at the same predictors (Deslee et al., 2023) found only depression to be significant.

As in other studies (Alcázar-Navarrete et al., 2022; Bogart et al., 2024), medication costs were lower in the SITT cohort than in the MITT cohort. The cost-effectiveness analysis results showed that SIIT was the dominant option. Similar results were found in a systematic review evaluating the cost-effectiveness of single versus multiple inhalers in asthma and COPD (Zhang et al., 2020). However, our region has more patients with MITT than with SITT. This may be because SITTs have been on the market for a shorter time than MITTs, and changes in prescribing habits occur gradually.

The limitations of our study are similar to those of other observational studies (Alcázar-Navarrete et al., 2022; Mannino et al., 2022; Deslee et al., 2023; Lin et al., 2023; Bogart et al., 2024). As in other studies published by our group (Pedrosa-Naudín et al., 2022; Gutiérrez-Abejón et al., 2023; 2024; Martín-Fernández et al., 2025), we assumed that dispensing approximates consumption. As in previous studies (Martín-Fernández et al., 2025), we excluded patients who had fewer than two prescription refills. This may be a confounding factor because not filling more prescriptions could be related to the perception that the treatment is ineffective. In this case, the number of patients excluded from each cohort was similar: 3,172 from the MITT group and 3,364 from the SITT group. Moderate exacerbations were extrapolated from pharmacy claims without physician confirmation. Due to a lack of access to hospital admission data, severe exacerbations could not be assessed. Finally, only drug-related costs were included in the cost-effectiveness analysis.

In conclusion, the SITT cohort showed a 33% increase in adherence, a 56% improvement in persistence, and a 35% reduction in the risk of moderate exacerbations compared to the MITT cohort. In addition, moderate exacerbations were associated with a 15% reduction in the likelihood of persistence. Lastly, this study confirms that SITT is the most cost-effective treatment for patients with moderate to severe COPD and a history of exacerbations. These findings support the SITT initiation, especially for naïve patients, offering a practical and effective strategy for optimizing COPD management.

## Data Availability

The data analyzed in this study is subject to the following licenses/restrictions: Restrictions apply to the availability of these data. Data were obtained from regional health authorities (Gerencia Regional de Salud (GRS)) and may be requested from conciertofco@saludcastillayleon.es. Requests to access these datasets should be directed to conciertofco@saludcastillayleon.es.
