# Supplementary material for: Single-versus multiple-inhaler triple therapy in patients with COPD in Spain: a retrospective cohort study comparing adherence, persistence, risk of exacerbations and economic outcomes
Source: Front Pharmacol. 2025 Sep 19;16:1642470. doi: 10.3389/fphar.2025.1642470 (PMC12491219; doi:10.3389/fphar.2025.1642470)
Supplement: Supplementary file 1 [file DataSheet1.docx]

Supplementary Material

# Supplementary Figures and Tables

## Supplementary Tables

**Supplementary Table 1**. Single- and multiple-inhaler triple therapy available in Spain.

| **Triple Therapy** | | | **ATC Code** | **Name** |  |
| --- | --- | --- | --- | --- | --- |
| MITT | LABA/ICS + LAMA | LABA/ICS | R03AK06 | Salmeterol and fluticasone |  |
|  |  |  | R03AK07 | Formoterol and budesonide |  |
|  |  |  | R03AK08 | Formoterol and beclometasone |  |
|  |  |  | R03AK10 | Vilanterol and fluticasone furoate |  |
|  |  |  | R03AK11 | Formoterol and fluticasone |  |
|  |  | LAMA | R03BB04 | Tiotropium bromide |  |
|  |  |  | R03BB05 | Aclidinium bromide |  |
|  |  |  | R03BB06 | Glycopyrronium bromide |  |
|  |  |  | R03BB07 | Umeclidinium bromide |  |
|  | LABA/LAMA + ICS | LABA/LAMA | R03AL03 | Vilanterol and umeclidinium bromide |  |
|  |  |  | R03AL04 | Indacaterol and glycopyrronium bromide |  |
|  |  |  | R03AL05 | Formoterol and aclidinium bromide |  |
|  |  |  | R03AL06 | Olodaterol and tiotropium bromide |  |
|  |  | ICS | R03BA01 | Beclometasone |  |
|  |  |  | R03BA02 | Budesonide |  |
|  |  |  | R03BA05 | Fluticasone |  |
|  |  |  | R03BA07 | Mometasone |  |
|  |  |  | R03BA08 | Ciclesonide |  |
| SITT | LABA/LAMA/ICS | | R03AL08 | Vilanterol, umeclidinium bromide and fluticasone furoate |  |
|  |  |  | R03AL09 | Formoterol, glycopyrronium bromide and beclometasone |  |
|  |  |  | R03AL11 | Formoterol, glycopyrronium bromide and budesonide |  |
| MITT, multiple-inhaler triple therapy; SITT, single-inhaler triple therapy; LABA, long-acting β2-agonists; LAMA, long-acting muscarinic antagonists; ICS, inhaled corticosteroids | | | | |  |
|  |  |  |  |  |  |

**Supplementary Table 2**. Baseline Characteristics of the Study Population.

|  | **Total** | **MITT** | **SITT** |
| --- | --- | --- | --- |
| **N** | 19791 | 12692 | 7099 |
| **Sociodemographic characteristics, N (%)** | |  |  |
| *Sex* |  |  |  |
| Male | 12482 (63.07) | 7677 (60.49) | 4805 (67.69) |
| *Age (mean±SD)* | 74.71±11.98 | 74.94±12.42 | 74.29±11.14 |
| *Distribution by age groups* |  |  |  |
| 40-49 | 580 (2.93) | 425 (3.35) | 154 (2.17) |
| 50-59 | 1617 (8.17) | 1085 (8.55) | 531 (7.48) |
| 60-69 | 4267 (21.56) | 2604 (20.52) | 1662 (23.41) |
| 70-79 | 5912 (29.87) | 3594 (28.32) | 2317 (32.64) |
| ≥ 80 | 7418 (37.48) | 4983 (39.26) | 2435 (34.3) |
| *Institutionalized* | 1031 (5.21) | 684 (5.39) | 347 (4.89) |
| *Healthcare Area* |  |  |  |
| Rural | 9789 (49.46) | 6239 (49.16) | 3550 (50.01) |
| Urban | 10002 (50.54) | 6453 (50.84) | 3549 (49.99) |
| *Socioeconomial level* |  |  |  |
| *< 18000 €/year* | 14598 (73.76) | 9431 (74.31) | 5165 (72.76) |
| *18000-100000 €/year* | 5112 (25.83) | 3210 (25.29) | 1903 (26.81) |
| *≥ 100000 €/year* | 73 (0.37) | 44 (0.35) | 30 (0.42) |
| **Clinical characteristics, N (%)** |  |  |  |
| *Naive patients* | 1878 (9.49) | 871 (6.86) | 1008 (14.2) |
| *Polypharmacy* | 18902 (95.51) | 12175 (95.93) | 6728 (94.77) |
| *Polypharmacy level* |  |  |  |
| *5-10* | 5474 (27.66) | 3603 (28.39) | 1871 (26.36) |
| *11-15* | 6252 (31.59) | 4092 (32.24) | 2159 (30.41) |
| *> 15* | 7178 (36.27) | 4480 (35.3) | 2698 (38.01) |
| *Multiple pharmacies* | 6521 (32.95) | 4270 (33.64) | 2253 (31.74) |
| *Multiple prescribers* | 14060 (71.04) | 8596 (67.73) | 5464 (76.97) |
| *Switching* | 1318 (6.66) | 873 (6.88) | 446 (6.28) |
| *Dose escalation* | 1967 (9.94) | 1503 (11.84) | 465 (6.55) |
| **Comorbidities, N (%)** |  |  |  |
| *Hypertension* | 12413 (62.72) | 7916 (62.37) | 4497 (63.35) |
| *Dyslipidemia* | 9797 (49.5) | 6133 (48.32) | 3664 (51.61) |
| *Anxiety* | 6367 (32.17) | 4061 (32) | 2306 (32.48) |
| *Asthma* | 5884 (29.73) | 4463 (35.16) | 1422 (20.03) |
| *Depression* | 5225 (26.4) | 3290 (25.92) | 1934 (27.24) |
| *Diabetes Mellitus* | 5156 (26.05) | 3178 (25.04) | 1977 (27.85) |
| *Ischaemic heart disease* | 4085 (20.64) | 2574 (20.28) | 1511 (21.28) |
| *Heart failure* | 4035 (20.39) | 2462 (19.4) | 1573 (22.16) |
| *Psychotic illness* | 1765 (8.92) | 1121 (8.83) | 645 (9.09) |
| *Dementia* | 273 (1.38) | 183 (1.44) | 90 (1.27) |
| MITT, multiple-inhaler triple therapy; SITT, single-inhaler triple therapy, SD, standard deviation | | | |

**Supplementary Table 3.** Treatment persistence for 12 months follow-up modifying the GAP between prescription refills at 90 days.

|  | **Total** | **MITT** | **SITT** | **p** |
| --- | --- | --- | --- | --- |
| **N** | 19791 | 12692 | 7099 |  |
|  |  |  |  |  |
| *Persistence (95% IC)* |  |  |  |  |
| *3 months* | 86.37 (85.89-86.85) | 84.42 (83.78-85.05) | 89.87 (89.17-90.57) | 0.001 |
| *6 months* | 74.06 (73.45-74.67) | 70.72 (69.93-71.51) | 80.03 (79.1-80.96) | 0.001 |
| *12 months* | 65.03 (64.37-65.69) | 61.53 (60.69-62.38) | 71.28 (70.23-72.33) | 0.001 |
| Treatment persistence (days) (mean±SD) | 281.31±122.81 | 271.43±127.23 | 298.98±112.34 | 0.001 |
| Cox proportional hazards model* |  |  |  |  |
| *HR (95% CI)* |  |  | 1.54 (1.46-1.63) | 0.001 |
| *Reference group for Cox proportional hazards model: SITT (multivariate analysis) | | |  |  |
| MITT, multiple-inhaler triple therapy, SITT, single-inhaler triple therapy, CI, confidence interval; SD, standard deviation; HR, hazard ratio | | | | |
|  |  |  |  |  |

## Supplementary Figures


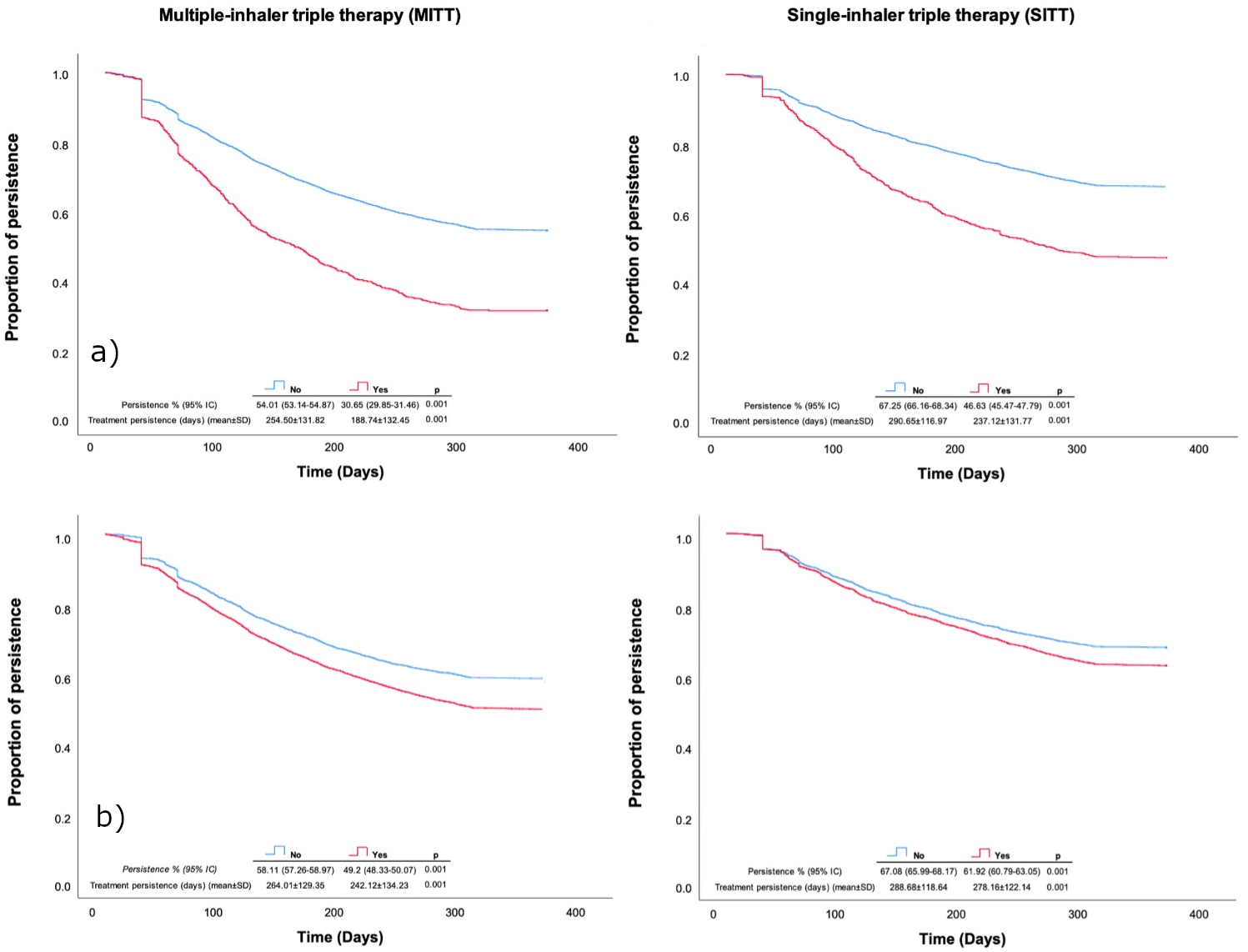


**Supplementary Figure 1.** Kaplan-Meier curve for treatment persistence in patients with COPD on single-inhaler triple therapy (SITT) and multiple-inhaler triple therapy (MITT) in Spain: a) naïve (Yes) vs. non-naive patients (No); b) patients with moderate exacerbations (Yes) vs. without moderate exacerbations (No).

.
